# Population data science: advancing the safe use of population data for public benefit

**DOI:** 10.4178/epih.e2018061

**Published:** 2018-12-25

**Authors:** Kerina Helen Jones, David Vincent Ford

**Affiliations:** Swansea University Medical School, Swansea, United Kingdom

**Keywords:** Data science, Big data, Medical informatics, International Population Data Linkage Network

## Abstract

The value of using population data to answer important questions for individual and societal benefit has never been greater. Governments and research funders world-wide are recognizing this potential and making major investments in data-intensive initiatives. However, there are challenges to overcome so that safe, socially-acceptable data sharing can be achieved. This paper outlines the field of population data science, the International Population Data Linkage Network (IPDLN), and their roles in advancing data-intensive research. We provide an overview of core concepts and major challenges for data-intensive research, with a particular focus on ethical, legal, and societal implications (ELSI). Using international case studies, we show how challenges can be addressed and lessons learned in advancing the safe, socially-acceptable use of population data for public benefit. Based on the case studies, we discuss the common ELSI principles in operation, we illustrate examples of a data scrutiny panel and a consumer panel, and we propose a set of ELSI-based recommendations to inform new and developing data-intensive initiatives.We conclude that although there are many ELSI issues to be overcome, there has never been a better time or more potential to leverage the benefits of population data for public benefit. A variety of initiatives, with different operating models, have pioneered the way in addressing many challenges. However, the work is not static, as the ELSI environment is constantly evolving, thus requiring continual mutual learning and improvement via the IPDLN and beyond.

## INTRODUCTION

Over the past two decades, we have witnessed a global explosion in the use of individual-level population data for research with technological advances and the rise of data-sharing infrastructures. This is creating previously unimaginable potential to integrate disparate sources of person-based data, to link them, and to make use of them for research, evaluation, and service planning. With the exception of Scandinavian countries [1], most data have been derived from health records, such as primary care and hospital data, with less availability of wider administrative data such as employment and education records. However, opportunities to integrate administrative data are growing, enabling exploration of wider factors that may influence health and well-being.

Many challenges must be addressed for this data-intensive work to be conducted in a safe, socially-acceptable manner. The purpose of this paper is to provide an overview of the core concepts and major issues in play, with a particular focus on ethical, legal, and societal implications (ELSI). It does this by using international examples as case studies to show how these challenges can be addressed and to present lessons learned. It draws upon the work of the International Population Data Linkage Network (IPDLN) and the unifying field of population data science in advancing data-intensive research.

The paper is organized as follows. We begin by setting the context for the work, defining concepts, identifying key ELSI requirements and challenges, and outlining the field of population data science and the IPDLN. We then provide a selection of international case studies with their various operating models to show how challenges can be addressed. From this, we draw material together to discuss successes, highlight outstanding challenges, and propose a set of ELSI-based recommendations to advance the safe use of population data for public benefit.

## APPROACH

Any work proposing to use individual-level person-based data for research must begin with a consideration of the legal context. This is essential to ensure there is a lawful justification for personal data processing, even if the data will ultimately be provided to researchers in anonymized form only. Within the European Union, the recent introduction of the General Data Protection Regulation (GDPR) [2] has overhauled data protection legislation in member states. However, it is important to note that the GDPR has wider relevance, since it applies anywhere in the world where data about European Union citizens are processed. It makes provisions for the lawful processing of general and special-category personal data. Usefully for research for public benefit, it sets out a justifiable lawful basis for such work as a task carried out in the public interest (Articles 6 and 9) [2]. We define public benefit in this paper as work with real-world value or practical applications with the clear potential to improve the lot of individuals and/or wider society [3]. Data protection legislation in South Korea is being reformed to promote safe data sharing, with policy-makers scrutinizing national laws and guidelines for better alignment with international trends [4]. The government is consolidating the national legislation, comprising the general Personal Information Protection Act of 2011 [5] and specific instruments, in a welcome move to advance opportunities for international collaborations.

**Figure 1.**
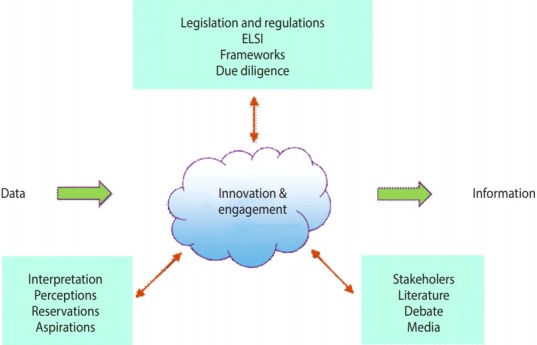
Illustration of the complex interplay between (1) legislative and regulatory frameworks; (2) the various interpretations, perceptions, and reservations that attend them; and (3) the debate, literature, and media coverage that ensue. These combine to create the complex, shifting space in which data-intensive initiatives operate. ELSI, ethical, legal, and societal implications.

Nonetheless, the safe use of person-based data for research requires far more than bare compliance with legislation, if it is to be perceived as ethical and socially-acceptable, since legality is not the same as social licence [6]. Many factors influence social perceptions in a complex, dynamic interplay. These must be taken into account when seeking to move forward, and there is a need for real innovation and public engagement in developing socially-acceptable data governance models (Figure 1).

Proper public engagement is essential to inform data-intensive research. Unlike research carried out face-to-face with participants, researchers may never meet the data subjects. This creates a disconnect and it is challenging to make engagement meaningful and reasonably representative. Some major initiatives are seeking to address this deficit, such as the Understanding Patient Data project of the UK Wellcome Trust [7]. This provides information in a digestible form, including short videos, so that members of the public can dip in and learn about how data are being used for research, with safeguards in place. A recently published international consensus paper sets out key principles of best practice in this important area. These include the need for transparency, inclusivity, and on-going engagement as a developmental process [8].

The field of population data science was developed following an extensive consultation with over 600 members of the IPDLN, to unite and consolidate the wealth of research and associated expertise surrounding data-intensive initiatives. It can be defined simply as ‘the science of data about people,’ and it incorporates under one umbrella all the disciplines that make safe, socially-acceptable data-intensive research possible and enable it to flourish. Its four characteristics are: data use for positive impact on citizens and society; bringing together and analysing data from multiple sources; finding population-level insights; and developing safe, privacy-sensitive, and ethical infrastructure to support research [9]. The IPDLN is a thriving international network, hosting a major biennial conference, and was the inspiration for the creation of the *International Journal of Population Data Science* (*IJPDS*, https://ijpds.org/index) which publishes articles on all aspects of research, development, and evaluation connected with population data.

**Figure 2.**
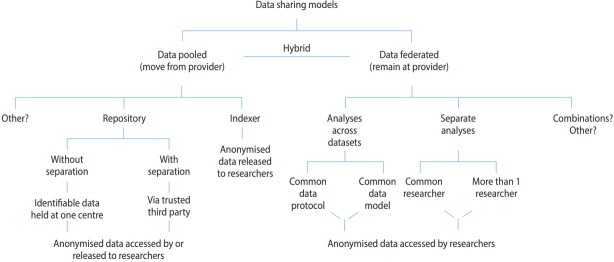
Illustration of some data-sharing models in operation in data-intensive initiatives. A basic dichotomy is shown between models where data are pooled or federated, but hybrid models also exist. As a general principle, data are provided to researchers in anonymized form.

Within the IPDLN, there are many organisations hosting major data-intensive initiatives with a variety of sharing models for the safe, socially-acceptable use of person-based data. We provide a selection of examples here to illustrate different approaches in use, since a detailed examination of all models is beyond the scope of this article. Models are illustrated in Figure 2, with the basic dichotomy being whether data are pooled or federated, which we define as data being accessed within a hosting organization or across source organisations, respectively.

Beginning with models where data are pooled, Population Data British Columbia (PopData) is an example of a data repository where the organization is authorized to hold data in identifiable form and to de-identify them for researcher access. This has been noted in Figure 2 as ‘without separation’ not to suggest that identifiable data are accessed in any unauthorized way, but to contrast this sort of model with those who need to use a trusted third party (TTP) to de-identify personal data before they can be received by the repository. PopData carries out data linkage and maintains a repository with a breadth of health and administrative datasets. It does not have its own research programme, but enables research by other parties, subject to approval. It operates a strictly-controlled, high-security environment with zones to enact separation principles and to control access in a privacy-by-design model. This term means that privacy is built into every aspect of the operating model. Researchers access their approved linked datasets within a secure virtual research environment. Researchers are prevented from downloading identifiable or row-level data. Instead, information for intended release is managed by PopData via an auditable process for transparency and accountability [10,11].

Similarly, the Institute of Clinical and Evaluative Sciences (IC/ES) in Ontario is an approved entity to collect, hold, and process personal health and administrative data and make datasets available to researchers within a secure research environment [12]. In contrast to PopData, IC/ES has its own extensive research programme with contracted researchers and a prolific set of research outputs (https://www.ices.on.ca/Publications).

The Secure Anonymised Information Linkage Databank (SAIL) in Wales (UK) is an example of a repository holding multiple health and wider administrative datasets that are linkable, but in a de-identified form. SAIL does not process person-identifiable data, but makes use of a TTP to carry out a matching and de-identification process, with the creation of a consistent, unique identifier for each person represented. A strict separation principle operates between the organisations involved. Data providers divide their data into two components: demographic and payload content. The demographic data are sent to the TTP and the payload data to SAIL. Recombination of de-identified datasets is performed at SAIL and the unique key allows linkage across datasets. Anonymised data are provided to researchers within a secure virtual environment and analysed remotely, subject to a suite of technical, physical, and procedural controls. Researchers are prevented from removing or altering the underlying data and the release of results is subject to scrutiny by a data guardian [13-15].

There is a long history of data-intensive initiatives in Australia. The Western Australian system is a long-standing example of an initiative that operates as an indexing centre, rather than hosting a data repository. Although the data are not gathered together at the organization, they are pooled as they are provided in de-identified linkable form to the researcher. The data centre maintains an index of links, enabling records to be requested from data providers and sent to researchers along with a project-linking key to join datasets arriving from different sources [16]. The Australian Population Health Research Network and the associated Centre for Data Linkage (CDL) provide researchers access to an array of state and federal health and administrative datasets (http://www.phrn.org.au/). Again, this operates on a sophisticated index-of-links model. The CDL holds demographic data to create an index of linkage keys, but does not have the content data. Linkage of federal-level (national) datasets is carried out by the CDL, whereas state-level linkage is conducted by units within those jurisdictions, such as the Centre for Health Record Linkage in Australian Capital Territory and New South Wales (http://www.cherel.org.au/).

Moving to examples of federated data models where data are not pooled, but remain at the data provider, we consider the provision of data for analyses across datasets and for separate analyses. The Canadian Network for Observational Drug Effect Studies (https://www.cnodes.ca/) comprises a distributed network of investigators and linked databases in British Columbia, Alberta, Saskatchewan, Manitoba, Ontario, Quebec and Nova Scotia. The basic operating model is that research questions are prioritised and developed within the geographical localities. The linked data remain within the provinces and a study team formulates a detailed protocol enabling constituent analyses in each province, from which results are returned. In such models, effective analyses across datasets may require a common data model with harmonization of data formats and structure, a common data protocol with a requirement for specifically structured queries, or may use both. A major development is underway for a National Data Platform to establish a single access portal connecting disparate provincial and pan-Canadian assets and capabilities using a hybrid repository and federated model.

Separate analyses of datasets are carried out in a simpler form of federated access in that they do not require common data models or protocols in order to function. However, this means they are more limited because of greater variability. A researcher accesses data held at two (or more) locations, carries out separate analyses, and then pools the results. As an alternative, depending on required approvals, it may be necessary for researchers employed by the host organization to access the data at each location and then share the results. This type of work usually occurs as a collaboration between repositories in different jurisdictions where the data cannot be pooled.

## DISCUSSION

The choice of the main data sharing model for a data-intensive initiative is subject to many factors, including the cost and availability of technical infrastructure [14]. Since ELSI issues are our particular interest, they will be the focus of this discussion. Beginning with legislation, it is sometimes the case that the movement of person-identifiable data is precluded by law. This is the case with certain UK government datasets; in order to access the data for research, a federated model needs to be used, unless a temporary legal gateway or a change in the law can be procured [17]. Ethical issues, such as the need to obtain informed consent, can also dictate how personal data can be used, unless authorization can be secured (as in the case of PopData and IC/ES), a waiver to consent can be obtained, or a suitable TTP can be engaged to manage the identifiable data (such as in the SAIL model). As well as statutory and regulatory issues, data provider permissions should also be considered. Providers must carry out due diligence processes depending on the status of their organization and the nature of the data of interest.

However, as well as complying with stipulated requirements, it is essential that data-intensive initiatives demonstrate trustworthiness to promote confidence in data providers and the public. This importance of this cannot be over-estimated, since trust takes time to gain, but can be easy to lose, resulting in major set-backs, as occurred with the English care.data initiative [6]. The 5 Safes constitute an example of a useful guiding framework for designing and evaluating data access models to demonstrate trustworthiness since they enable the opportunities, constraints, risks, and benefits of different approaches to be taken into account [18].

The 5 Safes are:

(1) Safe projects: Is this use of the data appropriate?

(2) Safe people: Can the researchers be trusted to use it in an appropriate manner?

(3) Safe data: Is there a disclosure risk in the data itself?

(4) Safe settings: Does the access facility limit unauthorised use?

(5) Safe outputs: Are the statistical results non-disclosive?

The models we have described each follow a guiding framework (such as the 5 Safes), although they may have differing mechanisms for doing so depending on their mode of operation, the nature of the data they work with, and the data protection landscape within their jurisdictions. Safe projects are commonly assessed via scrutiny panels that assess the appropriateness of the proposed data use. Having safe people is assured by accredited training courses for appropriate data use and initiatives to raise awareness of the repercussions of mis-use. Safe data and safe outputs are controlled by applying reliable measures to mitigate disclosure risk. Safe settings depend on a combination of technical, procedural, and physical controls on and around the data. Together, the overall package enacted by a data-intensive initiative comprises its privacy-by-design model, embodied in organizational policies that should be open to view and scrutiny.

We provide an example of the scrutiny panel in operation for the use of SAIL data. The Information Governance Review Panel (IGRP) is an independent group that assesses all proposals to use SAIL data before access can be granted to researchers [14]. Members include representatives of professional and regulatory bodies, and the general public. They are drawn from the British Medical Association, Public Health Wales, NHS Wales, Welsh Government, the National Research Ethics Service, and the Consumer Panel. The IGRP considers issues such as whether there is a clear and legitimate reason for conducting the project; whether all relevant approvals are in place (or being sought); and whether appropriate safeguards have been identified to address any potentially sensitive issues. To avoid unnecessary delays, the IGRP meets in a virtual space to review proposals and feedback is provided to researchers so that they can proceed or modify their work where needed.

The Consumer Panel (mentioned above) is an important part of SAIL data governance, and we consider their engagement with us to be indispensable in helping us demonstrate trustworthiness and transparency [19]. This active group of general public members meets quarterly and their role is to:

act as advisors on issues in research from the perspective of service users and carers; advise on how best to engage with the public/service users and carers; review information to be appropriate for the general reader; advise on how to recruit people to project steering groups; provide the service user/carer view on data protection issues; discuss proposals for projects and ideas for possible projects; act as advocates for data-intensive research

Other data-intensive initiatives have similar general public panels that provide invaluable advice and input for socially-acceptable conduct in the use of population data. Their commitment to public engagement is further demonstrated by the contributions of many international authors, from countries including the UK, Canada, Australia, the Netherlands, Finland, and Ireland, to the consensus statement [8].

To summarise this discussion, successful data-intensive initiatives are operating in many countries and using individual-level population data for research. Although their main models differ, common principles comprise their privacy-by-design models for the safe, socially-acceptable use of data. These centre on complying with all relevant legislative and regulatory requirements, but much more than that, they incorporate good practice mechanisms and engagement with stakeholders and the public to demonstrate trustworthiness. Based on this, we propose a set of recommendations to inform new and developing data-intensive initiatives.

### Recommendations

This set of recommendations relates to ELSI issues in working with individual-level population data, and is applicable for a variety data-intensive initiatives and models.

- Understand and comply with all relevant data protection legislation and regulations

- Select and operate a suitable framework to enable the safe use of data to be enacted and evaluated

- Develop a suite of transparent policies and procedures with clear responsibilities and accountabilities

- Provide clear and accessible information on how population data will be used

- Work closely with data providers and respect their due diligence processes

- Take public views into account, beyond the strict requirements of legislation, to promote inclusivity and social licence

- Support researchers in understanding and complying with their responsibilities for good conduct with data

- Enable the best-quality, most granular data to be used for research, without compromising privacy and security

- Acknowledge problems as soon as they arise and correct them as soon as possible

- Learn from other initiatives and share good practices as an on-going process to avoid ethical pitfalls

## CONCLUSION

This paper set out to provide an overview of core concepts and major challenges for data-intensive research, with a particular focus on ELSI issues. Although other aspects such as cost and technical infrastructure are essential considerations for data intensive-initiatives, ELSI issues must be addressed for the safe, socially-acceptable use of population data. Many advances have been made by member organisations of the IPDLN, and the new field of population data science is further consolidating and uniting the community across the world. Furthermore, the *IJPDS* provides a much-needed home for publications to promote and disseminate all aspects of population data science. We believe that there has never been a better time or more potential to leverage the benefits of population data for public benefit, with opportunities to learn from those who have pioneered the way in addressing key challenges. However, some challenges remain, and new ones arise as the ELSI environment evolves, requiring a process of continual learning from organisations in the IPDLN and more widely.
